# Effects of High Levels of Dietary Linseed Oil on the Growth Performance, Antioxidant Capacity, Hepatic Lipid Metabolism, and Expression of Inflammatory Genes in Large Yellow Croaker (*Larimichthys crocea*)

**DOI:** 10.3389/fphys.2021.631850

**Published:** 2021-02-17

**Authors:** Xueshan Li, Qiuchi Chen, Qingfei Li, Jiamin Li, Kun Cui, Yunqiang Zhang, Adong Kong, Yanjiao Zhang, Min Wan, Kangsen Mai, Qinghui Ai

**Affiliations:** ^1^Key Laboratory of Aquaculture Nutrition and Feed, Ministry of Agriculture and Rural Affairs, and the Key Laboratory of Mariculture, Ministry of Education, Ocean University of China, Qingdao, China; ^2^Laboratory for Marine Fisheries Science and Food Production Processes, Qingdao National Laboratory for Marine Science and Technology, Qingdao, China

**Keywords:** linseed oil, growth, antioxidant capacity, lipid metabolism, inflammation, *Larimichthys crocea*

## Abstract

A growth experiment was conducted to evaluate the effects of dietary fish oil (FO) replaced by linseed oil (LO) on the growth performance, antioxidant capacity, hepatic lipid metabolism, and expression of inflammatory genes in large yellow croaker (*Larimichthys crocea*). Fish (initial weight: 15.88 ± 0.14 g) were fed four experimental diets with 0% (the control), 33.3%, 66.7%, and 100% of FO replaced by LO. Each diet was randomly attributed to triplicate seawater floating cages (1.0 × 1.0 × 2.0 m) with 60 fish in each cage. Results showed that the growth performance of fish fed the diet with 100% LO was markedly decreased compared with the control group (*P* < 0.05), while no remarkable difference was observed in the growth performance of fish fed diets within 66.7% LO (*P* > 0.05). The percentage of 18:3n-3 was the highest in the liver and muscle of fish fed the diet with 100% LO among the four treatments. When dietary FO was entirely replaced by LO, fish had a markedly higher total cholesterol, total triglyceride, low-density lipoprotein cholesterol content, and alanine transaminase activity in the serum than the control group (*P* < 0.05). The concentration of malondialdehyde was markedly higher, while the activity of catalase was markedly lower in fish fed the diet with 100% LO than the control group (*P* < 0.05). When dietary FO was entirely replaced by LO, hepatic lipid content, transcriptional levels of *fatp1* and *cd36*, and CD36 protein expression were significantly higher, while transcriptional level of *cpt-1* and CPT-1 protein expression were significantly lower than the control group (*P* < 0.05). As for the gene expression of cytokines, fish fed the diet with 100% LO had markedly higher transcriptional levels of *il-1*β, *tnf*α, and *il-6* than the control group (*P* < 0.05). In conclusion, the substitution of 66.7% FO with LO had no significant effects on the growth performance of fish, while 100% LO decreased the growth performance and increased the inflammation and hepatic lipid content of fish. The increase of hepatic lipid content was probably due to the increased fatty acid uptake and decreased fatty acid oxidation in fish.

## Introduction

Linseed oil (LO) is rich in α-linoleic acid (18:3n-3), which is a vital fatty acid (FA) for fish to maintain good growth performance and health status (Menoyo et al., 2007; Wu and Chen, 2012). Furthermore, due to its relatively economical price and high production, LO is considered as a promising lipid source to replace fish oil (FO) in aquafeed (Turchini et al., 2009). Previous investigations have elucidated that LO can be effectively utilized by certain freshwater fishes; dietary FO could be partially replaced by LO in silver barb (*Puntius gonionotus*) (Nayak et al., 2017) or even entirely replaced by LO in murray cod (*Maccullochella peelii peelii*) (Turchini et al., 2011), rainbow trout (*Oncorhynchus mykiss*) (Filiz et al., 2017), and tilapia (*Oreochromis niloticus*) (Peng et al., 2016) without severely compromising the growth performance and health status. Nevertheless, when dietary FO was excessively replaced by LO, fish growth and health were reduced, and hepatic lipid deposition was increased in certain marine fishes, such as the yellow drum (*Nibea albiflora*) (Qin et al., 2020), turbot (*Scophthalmus maximus*) (Wang et al., 2016), and gilthead seabream (*Sparus aurata*) (Menoyo et al., 2004; Montero et al., 2008).

Lipid metabolism consists of lipid uptake, FA oxidation, lipid synthesis, and triglyceride-rich lipoprotein secretion (Musso et al., 2009). After the digestion and hydrolysis of dietary lipid, the circulating lipoproteins and non-esterified FAs are absorbed into hepatocytes through lipoprotein receptors, fatty acid transport protein 1 (*fatp1*) and cluster of differentiation 36 (*cd36*) (Stahl, 2004; Musso et al., 2009). After absorption, cytoplasmic FAs can be oxidized by carnitine palmitoyltransferase 1 (*cpt-1*) in mitochondria, and acyl CoA oxidase (*aco*) in peroxisomes, respectively (Ruyter et al., 1997; Mcgarry and Brown, 2010; Li et al., 2018). Furthermore, the acetyl CoA, produced by FA oxidation or other metabolic reactions, is an intermediate of the *de novo* lipogenesis. Fatty acid synthase (*fas*) and sterol-regulatory element-binding protein 1 (*srebp1*) represent a key lipogenic enzyme and a key transcription factor in lipid synthesis, respectively (Eberlé et al., 2004; Menendez and Lupu, 2007). However, when lipids are excessively synthesized in the cytoplasm, lipids can be excreted from the hepatocytes in the form of triglyceride-rich lipoproteins that are mediated by microsomal triacylglycerol transfer protein (*mtp*) and apolipoprotein B100 (*apoB100*) (Olofsson and BorÈN, 2005; Hussain et al., 2012). Thus, lipid metabolism is a useful approach to evaluate the utilization of vegetable oils in fish (Yan et al., 2015).

An imbalance in lipid accumulation (lipoprotein and FA uptake as well as lipid synthesis) and lipid consumption (FA oxidation and very-low-density lipoprotein secretion) can induce abnormal lipid deposition, which is always accompanied by inflammation in fish (Tan et al., 2017). Interleukin-1β (*il-1*β), interferon γ (*ifn*γ), cyclooxygenase-2 (*cox-2*), tumor necrosis factor α (*tnf*α), and interleukin-6 (*il-6*) are significant pro-inflammatory and chemotactic cytokines in fish (Pohlenz and Gatlin, 2014; Zou and Secombes, 2016). Meanwhile, interleukin-4 (*il-4*), interleukin-10 (*il-10*), and transforming growth factor β (*tgf*β) are deemed to be anti-inflammatory cytokines, and arginase-1 (*arg-1*) is deemed to be an anti-inflammatory enzyme. Those anti-inflammatory factors can be regulated by FAs to limit the over-activation of immune regulatory pathways (Kühn et al., 1993; Berg et al., 1995). Furthermore, the aforementioned lipid metabolism-related proteins and inflammatory factors can partly reflect the physiological state of fish (Montero et al., 2008; Xu et al., 2015). Nevertheless, few studies have systematically evaluated the lipid metabolism and the inflammation of fish that were fed diets with different LO levels.

Large yellow croaker (*Larimichthys crocea*) is a widely cultivated marine fish in China (Xiao et al., 2015; Chen et al., 2018; Huang et al., 2020). The appropriate use of LO in aquafeed can yield considerable benefits in terms of fish health, growth, and the economy (Wabike et al., 2020). Although a previous study has shown that passive effects were found in large yellow croaker when FO was entirely replaced by LO (Mu et al., 2020), the specific growth performance, lipid metabolism and inflammation in this fish fed diets with different LO levels still needs to be assessed to determine an appropriate substitution level of FO in the diet. Thus, the aim of this study was to evaluate the effects of dietary LO on the growth performance, antioxidant capacity, hepatic lipid metabolism, and inflammation in this fish for the further utilization of LO in aquafeed.

## Materials and Methods

### Diet Formulation

Four isonitrogenous (approximately 42% protein) and isolipidic (approximately 12% lipid) experimental diets were formulated to contain graded levels (0%, 33.3%, 66.7%, and 100%) of FO replaced by LO, and the FO group was regarded as the control (Table 1). Raw materials were pulverized into a fine powder and thoroughly mixed with water and oils to make diets (2.0 × 5.0 mm). Specific procedures of making diets and storing them were applied according to Ai et al. (2006). Four experimental diets were similar in quality and sinking properties, and FA composition of oils and diets was determined in this study (Table 2).

**TABLE 1:** Formulation and chemical proximate analysis of the experimental diets (% dry weight).

Ingredients	Fish oil replacement level/%			
	0%	33.3%	66.7%	100%
White fish meal^a^	35	35	35	35
Soybean meal^a^	28	28	28	28
Wheat meal^a^	23.8	23.8	23.8	23.8
Fish oil	7.5	5.0	2.5	0
Linseed oil	0	2.5	5.0	7.5
Soybean lecithin	1.5	1.5	1.5	1.5
Vitamin premix^b^	2	2	2	2
Mineral premix^b^	2	2	2	2
Attractant mixture^c^	0.1	0.1	0.1	0.1
Mold inhibitor^d^	0.1	0.1	0.1	0.1
Total	100	100	100	100
**Proximate analysis**				
Crude protein (%)	41.93	42.20	41.95	42.42
Crude lipid (%)	12.43	12.68	12.23	12.14
Gross energy (GE, MJ/Kg)	20.08	20.32	20.41	20.51
Protein/energy ratio (P/E ratio, g/MJ)	20.88	20.77	20.55	20.68

*^a^The composition of ingredients were determined by Li X. S. et al. (2019). ^b^The mixture of mineral mixture and vitamin mixture according to Yan et al. (2015). ^c^Attractant: the mixture of 50% glycine acid and 50% betaine by weight. ^d^Mold inhibitor: the mixture of 50% calcium propionic acid and 50% fumaric acid by weight.*

**TABLE 2:** Fatty acid composition of linseed oil and the experimental diets (% total fatty acids)^i^.

Fatty acid (% total fatty acids)	Fish oil^h^	Linseed oil^h^	Linseed oil replacement level/%			
			0%	33.3%	66.7%	100%
14:0	8.77	0.07	6.58	4.43	2.65	0.66
16:0	19.15	5.39	22.62	18.58	15.06	11.10
18:0	4.26	3.76	5.41	5.50	5.39	5.17
20:0	1.18	0.17	1.38	1.24	1.05	0.99
ΣSFA^a^	33.36	9.39	35.97	29.92	24.69	18.53
16:1n-7	11.85	0.10	8.81	6.21	3.70	1.22
18:1n-9	9.75	20.45	13.81	15.46	18.04	19.94
ΣMUFA^b^	21.60	20.55	22.63	21.67	21.74	21.17
18:2n-6 (LNA^c^)	1.54	15.52	10.76	13.32	16.44	18.76
20:4n-6	1.30	0.00	1.06	0.85	0.54	0.34
Σn-6PUFA^d^	2.84	15.52	11.82	14.17	16.97	19.10
18:3n-3 (ALA^e^)	0.76	53.02	1.43	10.89	20.88	30.23
20:5n-3	12.34	0.00	7.07	5.21	3.04	1.11
22:6n-3	7.30	0.00	5.15	4.15	2.71	1.70
Σn-3PUFA^f^	20.41	53.02	13.65	20.24	26.64	33.03
ALA/LNA	0.49	3.42	0.13	0.82	1.27	1.61
n-3/n-6PUFA	7.18	3.42	1.15	1.43	1.57	1.73
Σn-3LC-PUFA^g^	19.64	0.00	12.22	9.35	5.75	2.80

*^a^SFA, saturated fatty acids. ^b^MUFA, mono-unsaturated fatty acids. ^c^LNA, Linoleic acid. ^d^n-6 PUFA, n-6 poly-unsaturated fatty acids. ^e^ALA, α-linolenic acid. ^f^n-3 PUFA, n-3 poly-unsaturated fatty acids. ^g^LC-PUFA, long chain-polyunsaturated fatty acids. ^h^Fatty acid composition of fish oil and linseed oil is according to Li Y. N. et al. (2019) supplemental data. ^i^Some fatty acids, of which the percentages are minor, trace amount or not detected, such as 20:0, 22:0, 24:0, 14:1, 20:1n-9, 22:1n-11, 20:2n-6, 18:3n-6, 20:3n-6, 18:4n-3, and 22:5n-3, are not listed in the table.*

### Proximate Composition and FA Composition Determination

The crude protein, lipid, ash, and moisture of experimental diets and fish were determined according to the Association of Official Analytical Chemists (AOAC, 1995). Fish tissue was lyophilized to determine the moisture content, and the freeze-dried tissue was used to analyze the lipid content according to Folch et al. (1957). The gross energy content of experimental diets was analyzed by combustion in an oxygen bomb calorimeter PARR6400 (Parr Instrument Company, United States). Moreover, the FA composition of muscle and liver samples was analyzed according to Metcalfe et al. (1966) with a few modifications (Zuo et al., 2012). Specific procedures for determining FA composition were conducted according to Li X. S. et al. (2019) and Feng et al. (2017). The extracted FA methyl esters were detected by a gas chromatograph HP6890 (Agilent, Santa Clara, CA, United States).

### Experimental Procedures

L. crocea of the same batch were supplied by a local farm in Ningde, Fujian, China. Fish were cultured in floating cages (2 × 4 × 2.5 m) and adapted to the FO diet for 14 days. After the adaptation period, juveniles (initial weight: 15.88 ± 0.14 g) were randomly apportioned to 12 floating cages (1 × 1 × 2 m) with 60 juveniles in each cage. Each experimental diet was randomly allocated to three experimental cages. Juveniles were well fed twice daily (5:30 a.m. and 5:00 p.m.) to apparent visual satiation (when fish were not actively eating) for 10 weeks. This study was approved by the Institutional Animal Care and Use Committee of the Ocean University of China. The protocols for animal care and handling were strictly conducted according to Standard Operation Procedures (approval number: 20001001) of the Institutional Animal Care and Use Committee of the Ocean University of China.

### Sample Collection

At the end of the experiment, juveniles fasted for an entire day and were anesthetized with eugenol (Sigma-Aldrich, United States, 1:10,000) before sample collection (Mai et al., 2006). Subsequently, final fish numbers and total fish weight per cage were determined to calculate survival rate and growth performance. Four fish per cage were randomly collected for proximate composition analysis. The weight of the liver and visceral mass and the length of seven fish per cage were recorded to analyze morphological parameters. After then, the liver and muscle samples of the aforementioned seven fish were sampled to determine the proximate composition, FA composition, enzyme activity, and transcriptional level of genes. Blood was collected and placed at 4°C overnight to collect the serum after centrifugation (4°C, 4,000 × g for 10 min), and the serum was kept at −80°C until analysis.

### Biochemical Analysis

Total triglyceride (TG), total cholesterol (TC), high-density lipoprotein cholesterol (HDL-C), low-density lipoprotein cholesterol (LDL-C), activities of serum aspartate aminotransferase (AST), and alanine transaminase (ALT) were determined by an automatic chemistry analyzer (BS200, Mindray, China) using diagnostic reagent kits (Mindray Bio Medical Co., Ltd., China). The concentration of malondialdehyde (MDA), total antioxidant capacity (T-AOC), and activities of catalase (CAT) and superoxide dismutase (SOD) in the liver were detected using commercial reagent kits provided by Nanjing Jiancheng Bioengineering Institute (Nanjing, China).

### Real-Time Quantitative Polymerase Chain Reaction (RT-qPCR)

Three fish livers from the same cage were mixed into a tube, and the mixed samples were pulverized into powder in liquid nitrogen. RNA was isolated from 18 fish samples per group using TRIzol reagent (Takara, Japan) according to the manufacturer’s instructions. The DNA contaminant in isolated RNA was removed, and cDNA was synthesized with 1,000 ng of the isolated RNA using the PrimeScript^TM^ RT reagent Kit (Takara, Japan). The primer sequences were designed based on the nucleotide sequences of L. crocea (Table 3), where β-actin and glyceraldehyde 3-phosphate dehydrogenase (gapdh) were considered as house-keeping genes (Zheng et al., 2016). The volume of reaction system and the RT-qPCR program were performed according to Zuo et al. (2012) and Li X. S. et al. (2019). Transcriptional level of genes was analyzed using the 2^–Δ^ ^Δ^ ^Ct^ method (Livak and Schmittgen, 2001).

**TABLE 3:** Sequences of the PCR primers used in this study.

Target gene	Forward primers (5’–3’)	Reverse primers (5’–3’)	Accession number
aco	AGTGCCCAGATGATCTTGAAGC	CTGCCAGAGGTAACCATTTCCT	JX456348
apoB100	AGAGTGTTGTCCAGGATAAAGATGC	CAGGGCTCAGGGTCTCAGTC	KM593126
arg1	AACCACCCGCAGGATTACG	AAACTCACTGGCATCACCTCA	XM019269015
β-actin	GACCTGACAGACTACCTCATG	AGTTGAAGGTGGTCTCGTGGA	GU584189
cd36	GAGCATGATGGAAAATGGTTCAAAG	CTCCAGAAACTCCCTTTCACCTTAG	KM593122
cox-2	CTGGAAAGGCAACACAAGC	CGGTGAGAGTCAGGGACAT	KP259877
cpt-1	GCTGAGCCTGGTGAAGATGTTC	TCCATTTGGTTGAATTGTTTACTGTCC	JX434612
fas	CAGCCACAGTGAGGTCATCC	TGAGGACATTGAGCCAGACAC	JX456351
fatp1	CAACCAGCAGGACCCATTACG	CATCCATCACCAGCACATCACC	KM593124
gapdh	GACAACGAGTTCGGATACAGC	CAGTTGATTGGCTTGTTTGG	XM010743420
ifnγ	TCAGACCTCCGCACCATCA	GCAACCATTGTAACGCCACTTA	KM501500
il-1β	CATAGGGATGGGGACAACGA	AGGGGACGGACACAAGGGTA	KJ459927
il-4	AGTTCTTCTGTCGCGCTGAG	GCTATGTATGTGCGGTTGCTG	KU885453
il-10	AGTCGGTTACTTTCTGTGGTG	TGTATGACGCAATATGGTCTG	XM010738826
il-6	CGACACACCCACTATTTACAAC	TCCCATTTTCTGAACTGCCTCT	KU140675
mtp	ATGTCCAAAATGTTCTCCATGTCTG	ATGTCAATAGCCAACCCTCCTTG	KP027412
srebp1	TCTCCTTGCAGTCTGAGCCAAC	TCAGCCCTTGGATATGAGCCT	KP342262
tgfβ	AGCAACCACCGTACATCCTG	AGGTATCCCGTTGGCTTGTG	XM027280465
tnfα	ACACCTCTCAGCCACAGGAT	CCGTGTCCCACTCCATAGTT	EF165623

*aco, acyl-CoA oxidase; apoB100, apolipoprotein B100; arg-1, arginase-1; cd36, cluster of differentiation 36; cox-2, cyclooxygenase-2; cpt-1, carnitine palmitoyltransferase 1; fas, fatty acid synthase; fatp1, fatty acid transport protein 1; gapdh, glyceraldehyde-3-phosphate dehydrogenase; hl, hepatic lipase; ifnγ, interferon γ; il-1β, interleukin-1β; il-4, interleukin-4; il-10, interleukin-10; il-6, interleukin-6; mtp, microsomal triacylglycerol transfer protein; srebp1, sterol-regulatory element-binding protein 1; tgfβ, transforming growth factor β; tnfα, tumor necrosis factor α.*

### Western Blot

Total protein of hepatic samples was extracted by RIPA reagent (Beyotime Biotechnology, China) according to the methods described by Xu et al. (2016). Protein concentration was quantified by a BCA Protein Assay Kit (Solarbio, China). The specific procedures of western blot were conducted according to Li X. S. et al. (2019). The GAPDH was used as the reference (Cui et al., 2020). Antibody against MTP (ab186446) was purchased from Abcam (England). Antibody against CPT-1 (#15184-1-AP) was purchased from Proteintech (United States). Antibody against CD36 was made by Genscript (China) via immunizing two adult New Zealand rabbits with antigen polypeptide (sequence: KGPYTYRTRYLPKEC) dissolved with complete Freund’s adjuvant. The validation of CD36 antibody was confirmed according to the methods described by Uhlen et al. (2016). Antibody against GAPDH (#TA-08) was purchased from Golden Bridge Biotechnology (China), and HRP-conjugated secondary antibody (#A0208) was purchased from Beyotime Biotechnology (China).

### Calculations and Statistical Methods

The following parameters were calculated:

$$Survivalrate\left(SR\%\right)=\frac{N_{F}}{N_{I}}\times 100$$

$$Specificgrowthrate\left(SGR,\%/d\right)=\frac{\left(LnW_{F}-LnW_{I}\right)}{t}\times 100$$

$$Feedintake\left(FI,\%/d\right)=\frac{F_{C}}{\left(\frac{W_{F}+W_{I}}{2}\right)\times t}\times 100$$

$$Feedefficiencyratio\left(FER\right)=\frac{W_{G}}{F_{C}}$$

$$Hepatosomaticindex\left(HSI\%\right)=\frac{W_{L}}{W_{F}}\times 100$$

$$Viscerosomaticindex\left(VSI\%\right)=\frac{W_{V}}{W_{F}}\times 100$$

$$Conditionfactor\left(CF\%\right)=\frac{W_{F}}{L_{F}^{3}}\times 100$$

where N_*I*_ and N_*F*_ represent the initial and final fish number, respectively; W_*I*_ and W_*F*_ represent the initial and final fish body weight, respectively; t represents the duration of experimental days; F_*C*_ represents the dry feed consumption in g; W_*G*_ represents the wet weight gain in g; W_*L*_ represents the hepatic wet weight; W_*V*_ represents the visceral wet weight; and L_*F*_ represents the fish body length.

Statistical analyses were processed by one-way analysis of variance (ANOVA) and then carried out Tukey’s test using SPSS 21.0 (IBM, United States) (He et al., 2019; Li S. L. et al., 2019). The column charts were made using GraphPad Prism 5 (GraphPad Software, Inc., La Jolla, CA, United States). The significance of statistics was judged by *P* < 0.05, and the results were represented as means ± S.E.M (standard error of the mean).

## Results

### Survival, Growth, and Morphological Parameters

Dietary LO levels had no remarkable difference in the SR of fish among the four treatments (*P* > 0.05). Compared with the control group, fish fed the diet with 100% LO showed markedly lower SGR and FER (*P* < 0.05). However, no remarkable differences were observed in the SGR and FER of fish fed diets within 66.7% LO (*P* > 0.05). Furthermore, there were no remarkable differences in the FI, VSI, HSI, or CF of fish among the four treatments (*P* > 0.05) (Table 4).

**TABLE 4:** Growth, survival, and morphological parameters of large yellow croaker fed diets with different levels of linseed oil (Means ± S.E.M)^a^.

Index	0%	33.3%	66.7%	100%
Initial body weight (IBW, g)	15.89 ± 0.08	15.87 ± 0.32	15.87 ± 0.42	15.91 ± 0.41
Final body weight (FBW, g)	37.67 ± 1.07^a^	36.71 ± 0.18^a^	35.92 ± 0.93^a^	32.03 ± 0.51^b^
Specific growth rate (SGR,%/d)	1.23 ± 0.05^a^	1.20 ± 0.04^ab^	1.17 ± 0.06^ab^	1.00 ± 0.04^b^
Survival rate (SR,%)	91.67 ± 1.67	88.33 ± 2.55	86.11 ± 1.47	84.44 ± 1.11
Feed Intake (FI,%/d)	2.11 ± 0.08	2.29 ± 0.11	1.96 ± 0.08	2.22 ± 0.01
Feed efficiency ratio (FER)	0.55 ± 0.02^a^	0.50 ± 0.03^ab^	0.56 ± 0.01^a^	0.43 ± 0.02^b^
Viscera-somatic index (VSI,%)	7.12 ± 0.26	6.71 ± 0.33	6.79 ± 0.33	7.00 ± 0.37
Hepato-somatic index (HSI,%)	1.96 ± 0.15	1.93 ± 0.10	2.07 ± 0.11	2.19 ± 0.13
Condition Factor (CF,%)	0.99 ± 0.03	1.00 ± 0.02	1.06 ± 0.04	1.00 ± 0.02

*^a^Data are presented as means ± S.E.M. Means in each row sharing the same superscript letter or absence of superscripts are not significantly different determined by Tukey’s test (P > 0.05). S.E.M., standard error of means.*

### Body Composition Analysis

When dietary FO was entirely replaced by LO, fish had markedly higher hepatic lipid content than the control group (*P* < 0.05). However, there were no remarkable differences in the lipid or moisture contents of the muscle as well as the moisture, lipid, protein, or ash contents of the whole body among the four treatments (*P* > 0.05) (Table 5).

**TABLE 5:** Body composition analysis of large yellow croaker fed diets with different levels of linseed oil (Means ± S.E.M)^a^.

Index (wet weight,%)	0%	33.3%	66.7%	100%
**Whole body (%)**				
Moisture	72.26 ± 0.73	72.32 ± 0.29	72.95 ± 1.08	72.14 ± 0.60
Lipid	7.63 ± 0.22	7.39 ± 0.18	7.22 ± 0.10	7.61 ± 0.18
Protein	15.48 ± 0.36	16.20 ± 0.41	15.94 ± 0.28	16.45 ± 0.32
Ash	3.89 ± 0.14	3.91 ± 0.23	4.19 ± 0.17	3.87 ± 0.16
**Liver (%)**				
Moisture	59.10 ± 0.72	61.85 ± 0.68	64.53 ± 4.08	57.87 ± 1.25
Lipid	22.79 ± 1.28^b^	26.20 ± 1.08^b^	27.55 ± 1.43^ab^	32.07 ± 1.84^a^
**Muscle (%)**				
Moisture	73.82 ± 0.61	72.43 ± 0.96	73.22 ± 1.77	76.06 ± 0.63
Lipid	7.54 ± 0.53	8.05 ± 0.60	8.44 ± 0.46	9.29 ± 0.45

*^a^Data are presented as means ± S.E.M. Means in each row sharing the same superscript letter or absence of superscripts are not significantly different determined by Tukey’s test (P > 0.05). S.E.M., standard error of means.*

### FA Composition in the Liver and Muscle

The percentage of 18:3n-3 (α-linolenic acid, ALA) was the highest in the liver and muscle of fish fed the diet with 100% LO among the four treatments (Tables 6, 7). However, when dietary FO was entirely replaced by LO, the hepatic percentage of ALA was lower than the hepatic percentage of 18:1n-9 (oleic acid, OA) in fish (Table 6). Moreover, percentages of OA and 18:2n-6 (linoleic acid, LNA) were significantly increased, while percentages of 14:0, 16:0, 16:1n-7, 20:4n-6 (arachidonic acid, ARA), 20:5n-3 (eicosapentaenoic acid, EPA), and 22:6n-3 (docosahexenoic acid, DHA) were significantly decreased in the liver and muscle of fish fed the diet with 100% LO compared with the control group (*P* < 0.05) (Tables 6, 7).

**TABLE 6:** Fatty acid composition (% total fatty acids) in the liver of large yellow croaker fed diets with different levels of linseed oil (Means ± S.E.M)^f^.

Fatty acid (% total fatty acids)	0%	33.3%	66.7%	100%
14:0	4.29 ± 0.36^a^	3.86 ± 0.33^ab^	2.90 ± 0.23^b^	1.33 ± 0.13^c^
16:0	22.28 ± 0.38^a^	20.49 ± 0.56^a^	17.55 ± 0.66^b^	13.96 ± 0.78^c^
18:0	7.14 ± 0.82	6.66 ± 0.08	6.16 ± 0.52	5.91 ± 0.52
20:0	1.62 ± 0.20	1.52 ± 0.12	1.56 ± 0.06	1.32 ± 0.14
ΣSFA^a^	35.32 ± 0.88^a^	32.52 ± 0.73^a^	28.17 ± 0.60^b^	22.51 ± 1.29^c^
16:1n-7	13.26 ± 0.43^a^	9.99 ± 0.46^b^	7.76 ± 1.16^bc^	4.76 ± 0.45^c^
18:1n-9	22.34 ± 1.56^b^	25.31 ± 1.26^ab^	27.62 ± 1.31^ab^	29.06 ± 1.37^a^
ΣMUFA^b^	35.60 ± 1.21	35.30 ± 1.31	35.38 ± 2.38	33.82 ± 1.81
18:2n-6	7.81 ± 0.73^b^	10.08 ± 1.52^ab^	12.89 ± 0.55^a^	14.00 ± 1.23^a^
20:4n-6	0.46 ± 0.01^a^	0.31 ± 0.10^ab^	0.13 ± 0.02^b^	0.13 ± 0.00^b^
Σn-6PUFA^c^	8.27 ± 0.72^b^	10.38 ± 1.61^ab^	13.02 ± 0.57^ab^	14.13 ± 1.23^a^
18:3n-3	1.42 ± 0.20^d^	7.14 ± 0.42^c^	14.77 ± 0.48^b^	20.48 ± 1.29^a^
20:5n-3(EPA)	3.80 ± 0.29^a^	1.96 ± 0.17^b^	1.04 ± 0.25^bc^	0.33 ± 0.06^c^
22:6n-3(DHA)	2.29 ± 0.35^a^	1.35 ± 0.11^b^	0.71 ± 0.11^bc^	0.39 ± 0.02^c^
Σn-3PUFA^d^	7.52 ± 0.41^c^	10.45 ± 0.26^c^	16.52 ± 0.67^b^	21.20 ± 1.31^a^
n-3/n-6PUFA	0.92 ± 0.09	1.06 ± 0.16	1.28 ± 0.09	1.54 ± 0.21
Σn-3LC-PUFA^e^	6.09 ± 0.42^a^	3.31 ± 0.17^b^	1.75 ± 0.20^c^	0.73 ± 0.04^c^

*^a^SFA, saturated fatty acids. ^b^MUFA, mono-unsaturated fatty acids. ^c^n-6 PUFA, n-6 poly-unsaturated fatty acids. ^d^n-3 PUFA, n-3 poly-unsaturated fatty acids. ^e^LC-PUFA, long chain-polyunsaturated fatty acids. ^f^Some fatty acids, of which the percentages are minor, trace amount or not detected, such as 20:0, 22:0, 24:0, 14:1, 20:1n-9, 22:1n-11, 20:2n-6, 18:3n-6, 20:3n-6, 18:4n-3 and 22:5n-3, are not listed in the table. Data are presented as means ± S.E.M. Means in each row sharing the same superscript letter or absence of superscripts are not significantly different determined by Tukey’s test (P > 0.05). S.E.M., standard error of means.*

**TABLE 7:** Fatty acid composition (% total fatty acids) in the muscle of large yellow croaker fed diets with different levels of linseed oil (Means ± S.E.M)^f^.

Fatty acid (% total fatty acids)	0%	33.3%	66.7%	100%
14:0	4.52 ± 0.12^a^	3.80 ± 0.04^b^	2.52 ± 0.10^c^	1.54 ± 0.18^d^
16:0	21.40 ± 0.34^a^	19.39 ± 0.57^b^	17.26 ± 0.33^c^	15.28 ± 0.18^d^
18:0	4.73 ± 0.21	4.69 ± 0.17	4.42 ± 0.20	4.06 ± 0.30
20:0	1.49 ± 0.04^a^	1.49 ± 0.04^a^	1.38 ± 0.08^ab^	1.16 ± 0.06^b^
ΣSFA^a^	32.13 ± 0.59^a^	29.37 ± 0.68^b^	25.58 ± 0.17^c^	22.04 ± 0.46^d^
16:1n-7	9.10 ± 0.35^a^	7.16 ± 0.22^b^	5.26 ± 0.04^c^	3.20 ± 0.18^d^
18:1n-9	15.26 ± 0.22^b^	16.72 ± 0.35^ba^	18.12 ± 1.04^a^	19.40 ± 0.49^a^
ΣMUFA^b^	24.36 ± 0.21	23.89 ± 0.36	23.38 ± 1.02	22.59 ± 0.46
18:2n-6	9.68 ± 0.28^c^	11.51 ± 0.31^bc^	13.68 ± 0.88^ab^	15.08 ± 0.22^a^
20:4n-6	0.94 ± 0.05^a^	0.83 ± 0.04^a^	0.58 ± 0.05^b^	0.58 ± 0.07^b^
Σn-6PUFA^c^	10.63 ± 0.33^c^	12.34 ± 0.32^bc^	14.26 ± 0.84^ab^	15.66 ± 0.28^a^
18:3n-3	1.45 ± 0.02^d^	9.13 ± 0.28^c^	17.34 ± 0.66^b^	25.80 ± 0.30^a^
20:5n-3(EPA)	6.62 ± 0.33^a^	5.28 ± 0.33^b^	3.34 ± 0.27^c^	1.54 ± 0.21^d^
22:6n-3(DHA)	5.66 ± 0.38^a^	4.52 ± 0.28^a^	3.07 ± 0.21^b^	2.01 ± 0.27^b^
Σn-3PUFA^d^	13.73 ± 0.69^d^	18.94 ± 0.52^c^	23.75 ± 0.64^b^	29.34 ± 0.39^a^
n-3/n-6PUFA	1.30 ± 0.09^b^	1.54 ± 0.06^ab^	1.68 ± 0.14^ab^	1.88 ± 0.06^a^
Σn-3LC-PUFA^e^	12.27 ± 0.68^a^	9.81 ± 0.43^b^	6.41 ± 0.34^c^	3.55 ± 0.10^d^

*^a^SFA, saturated fatty acids. ^b^MUFA, mono-unsaturated fatty acids. ^c^n-6 PUFA, n-6 poly-unsaturated fatty acids. ^d^n-3 PUFA, n-3 poly-unsaturated fatty acids. ^e^LC-PUFA, long chain-polyunsaturated fatty acids. ^f^Some fatty acids, of which the percentages are minor, trace amount or not detected, such as 20:0, 22:0, 24:0, 14:1, 20:1n-9, 22:1n-11, 20:2n-6, 18:3n-6, 20:3n-6, 18:4n-3, and 22:5n-3, are not listed in the table. Data are presented as means ± S.E.M. Means in each row sharing the same superscript letter or absence of superscripts are not significantly different determined by Tukey’s test (P > 0.05). S.E.M., standard error of means.*

### Serum Biochemical Parameters

The activity of ALT and contents of TC, TG, and LDL-C were markedly higher in fish fed the diet with 100% LO than the control group (*P* < 0.05). However, compared with the control group, the activity of serum ALT and contents of TC, TG, and LDL-C showed no remarkable difference in fish fed diets within 66.7% LO (*P* > 0.05). No remarkable differences were observed in the content of HDL-C or the activity of AST of fish among the four treatments (*P* > 0.05) (Table 8).

**TABLE 8:** Serum biochemical indexes and enzyme activities of large yellow croaker fed diets with different levels of linseed oil (Means ± S.E.M.)^g^.

Plasma biochemical indexes	0%	33.3%	66.7%	100%
TC^a^ (mmol/L)	2.67 ± 0.18^b^	2.86 ± 0.24^ab^	2.70 ± 0.13^b^	3.55 ± 0.23^a^
TG^b^ (mmol/L)	3.18 ± 0.22^b^	3.69 ± 0.43^b^	4.43 ± 0.30^ab^	5.26 ± 0.29^a^
HDL-C^c^ (mmol/L)	0.79 ± 0.03	0.77 ± 0.07	0.67 ± 0.05	0.59 ± 0.06
LDL-C^d^ (mmol/L)	0.75 ± 0.05^b^	0.82 ± 0.11^b^	0.83 ± 0.03^b^	1.15 ± 0.09^a^
ALT^e^ (U/L)	55.68 ± 3.13^b^	59.02 ± 3.74^ab^	62.99 ± 3.40^ab^	70.06 ± 2.66^a^
AST^f^ (U/L)	5.58 ± 0.48	5.95 ± 0.38	6.69 ± 0.38	7.17 ± 0.50

*^a^TC, Total cholesterol. ^b^TG, Total triglyceride. ^c^HDL-C, High-density lipoprotein cholesterol. ^d^LDL-C, Low-density lipoprotein cholesterol. ^e^ALT, Alanine transaminase. ^f^AST, Aspartate aminotransferase. ^g^Data are presented as means ± S.E.M. Means in each row sharing the same superscript letter or absence of superscripts are not significantly different determined by Tukey’s test (P > 0.05). S.E.M., standard error of means.*

### Antioxidant Capacity

**FIGURE 1:**
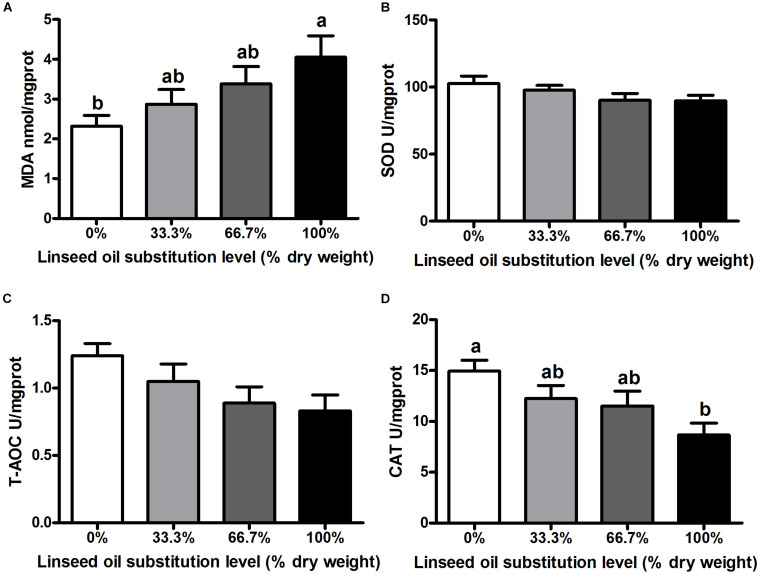
Concentration of **(A)** malondialdehyde (MDA) and activities of **(B)** superoxide dismutase (SOD), **(C)** total antioxidant capacity (T-AOC), and **(D)** catalase (CAT) in the liver of large yellow croaker. 0%: control diet with 0% fish oil replaced by linseed oil; 33.3%: diet with 33.3% fish oil replaced by linseed oil; 66.7%: diet with 66.7% fish oil replaced by linseed oil; 100%: diet with fish oil totally replaced by linseed oil; Data are presented as means ± S.E.M. Columns sharing the same superscript letter or absence of superscripts are not significantly different determined by Tukey’s test (P > 0.05). S.E.M., standard error of means.

When dietary FO was entirely replaced by LO, fish had the lower activity of CAT and the higher concentration of MDA in the liver than the control group (*P* < 0.05) (Figures 1A,D). However, no remarkable differences were observed in the activity of CAT and the concentration of MDA in fish fed diets within 66.7% LO (*P* > 0.05). Furthermore, there were no remarkable differences observed in activities of SOD or T-AOC of fish among the four treatments (*P* > 0.05) (Figures 1B,C).

### Expression of Lipid Metabolism-Related Genes

**FIGURE 2:**
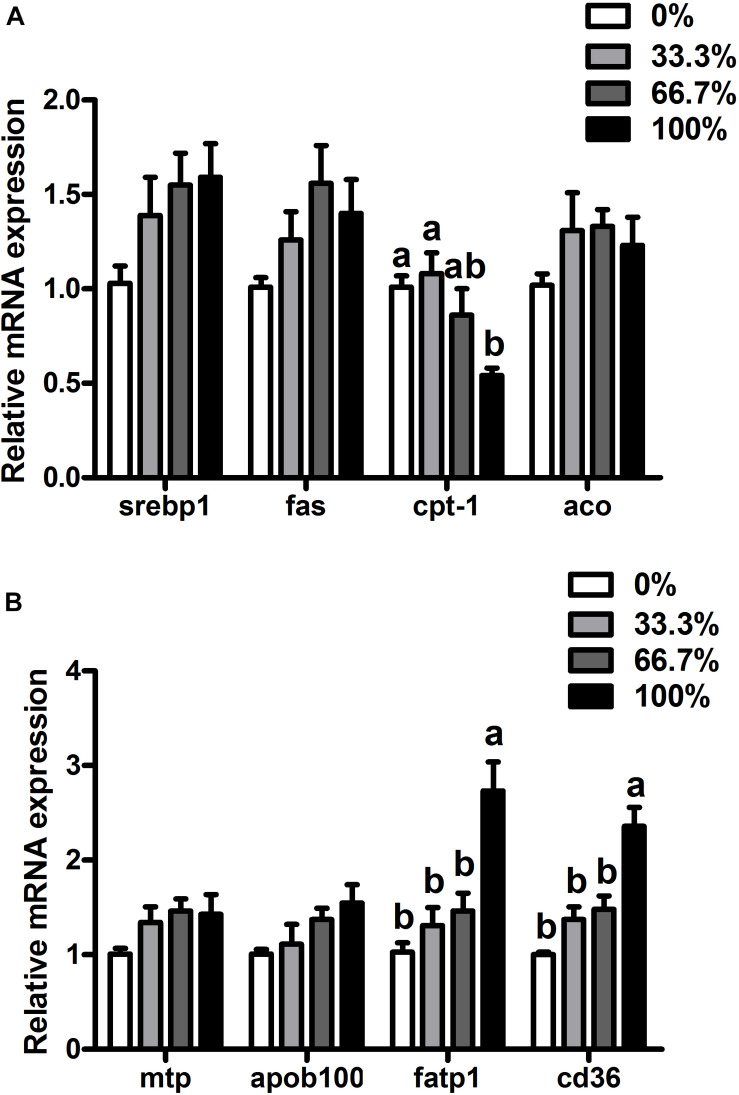
Expression of genes related to lipid metabolism in the liver of large yellow croaker. **(A)** *srebp1*, sterol-regulatory element-binding protein 1; *fas*, fatty acid synthase; *cpt-1*, carnitine palmitoyltransferase 1; *aco*, acyl-CoA oxidase; **(B)** *mtp*, microsomal triglyceride transfer protein; *apoB100*, apolipoprotein B100; *fatp1*, fatty acid transport protein 1; *cd36*, cluster of differentiation 36; 0%: control diet with 0% fish oil replaced by linseed oil; 33.3%: diet with 33.3% fish oil replaced by linseed oil; 66.7%: diet with 66.7% fish oil replaced by linseed oil; 100%: diet with fish oil totally replaced by linseed oil; Data are presented as means ± S.E.M. Columns sharing the same superscript letter or absence of superscripts are not significantly different determined by Tukey’s test (*P* > 0.05). S.E.M., standard error of means.

When dietary FO was entirely replaced by LO, fish had higher transcriptional levels of *fatp1* and *cd36* and a lower transcriptional level of *cpt-1* than the control group (*P* < 0.05). There were no remarkable differences in transcriptional levels of *srebp1*, *fas*, *aco*, *mtp*, or *apoB100* of fish among the four treatments (*P* > 0.05) (Figure 2).

### Expression of MTP, CPT-1 and CD36 Proteins

**FIGURE 3:**
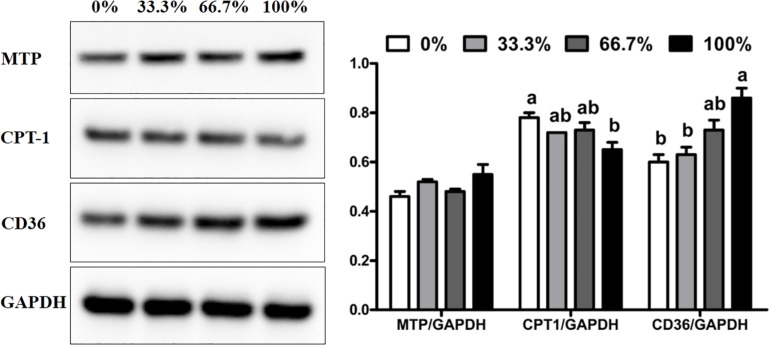
Expression of MTP, CD36, and CPT-1 proteins in the liver of large yellow croaker. MTP, microsomal triglyceride transfer protein; CPT-1, carnitine palmitoyltransferase 1; CD36, cluster of differentiation 36; GAPDH: glyceraldehyde-3-phosphate dehydrogenase. Data are expressed as A.U. of the western blot and are depicted as a ratio of MTP to GAPDH, CPT-1 to GAPDH, and CD36 to GAPDH. 0%: control diet with 0% fish oil replaced by linseed oil; 33.3%: diet with 33.3% fish oil replaced by linseed oil; 66.7%: diet with 66.7% fish oil replaced by linseed oil; 100%: diet with fish oil totally replaced by linseed oil; Data are presented as means ± S.E.M. Columns sharing the same superscript letter or absence of superscripts are not significantly different determined by Tukey’s test (*P* > 0.05). S.E.M., standard error of means.

When dietary FO was entirely replaced by LO, fish had the higher expression of CD36 protein and lower expression of CPT-1 protein than the control group (*P* < 0.05). There was no remarkable difference in the expression of CPT-1 and CD36 proteins in fish fed diets within 66.7% LO (*P* > 0.05). No remarkable difference was observed in the expression of MTP protein of fish among the four treatments (*P* > 0.05) (Figure 3).

### Expression of Pro-inflammatory and Anti-inflammatory Genes

**FIGURE 4:**
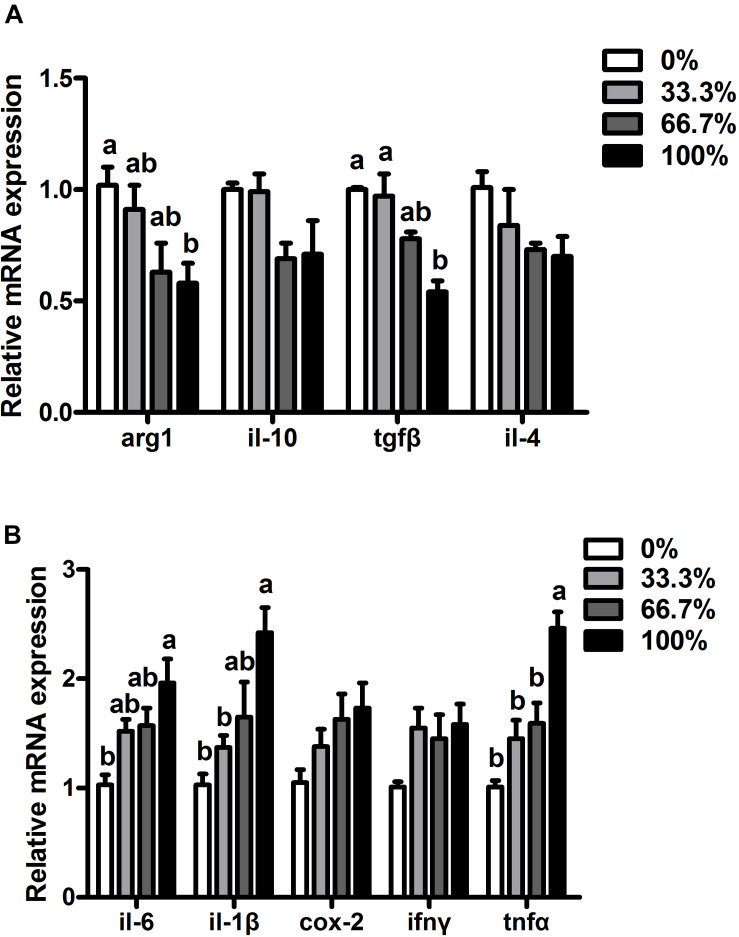
Expression of genes related to inflammation in the liver of large yellow croaker. **(A)** *arg-1*, arginase-1; *il-10*, interleukin-10; *tgf*β, transforming growth factor β; *il-4*, interleukin-4; **(B)** *il-6*, interleukin-6; *il-1*β, interleukin-1β; *cox-2*, cyclooxygenase-2; *ifnγ*, interferon *γ*; *tnf*α, tumor necrosis factor α; 0%: control diet with 0% fish oil replaced by linseed oil; 33.3%: diet with 33.3% fish oil replaced by linseed oil; 66.7%: diet with 66.7% fish oil replaced by linseed oil; 100%: diet with fish oil totally replaced by linseed oil; Data are presented as means ± S.E.M. Columns sharing the same superscript letter or absence of superscripts are not significantly different determined by Tukey’s test (*P* > 0.05). S.E.M., standard error of means.

When dietary FO was entirely replaced by LO, fish had higher transcriptional levels of pro-inflammatory genes (*il-6*, *il-1*β, and *tnf*α) and lower transcriptional levels of anti-inflammatory genes (*arg1* and *tgf*α) than the control group (*P* < 0.05). Compared with the control group, transcriptional levels of *il-6*, *il-1*β, *tnf*α, *arg1*, and *tgf*β showed no remarkable difference in fish fed diets within 66.7% LO (*P* > 0.05). No remarkable differences were found at the transcriptional levels of *ifnγ*, *cox-2*, *il-10*, or *il-4* of fish among the four treatments (*P* > 0.05) (Figures 4A,B).

## Discussion

In this study, the growth performance was compromised in *L. crocea* fed the diet with 100% LO, which was consistent with results found in turbot (Wang et al., 2016) and gilthead seabream (Montero et al., 2008). The decreased growth performance of *L. crocea* was probably due to the increase of ALA/LNA ratio and the decrease of n-3 LC-PUFA content with the increasing inclusion of LO. Evidence in the literature has shown that an appropriate ratio of ALA/LNA in diets can increase the growth performance of yellow catfish (*Pelteobagrus fulvidraco*) (Tan et al., 2009) and Atlantic salmon (*Salmo salar*). However, a high ratio of ALA/LNA in diets showed passive effects on the growth performance of tilapia (Li et al., 2016) and grass carp (*Ctenopharyngodon idellus*) (Zeng et al., 2016). In this study, the ratio of ALA/LNA was increased by 12.38 times in the 100% LO diet compared with that in the FO diet. In addition, n-3 LC-PUFA was deemed to be essential for marine fish, Zuo et al. (2012) have reported that the requirement of n-3 LC-PUFA for *L. crocea* is about 0.60–0.98% to maintain good growth performance and non-specific immunity. In this study, the content of n-3 LC-PUFA was approximately 0.34% in the 100% LO diet, which did not meet the requirement of n-3 LC-PUFA for *L. crocea*.

Besides the growth performance, the physiological state of fish has also become a research hotspot for nutritionists to evaluate lipid sources. Serum biochemical indices were useful terms to evaluate the physiological state of fish (Shi Y. et al., 2019). In this study, contents of serum TC, TG, and LDL-C were increased in fish when dietary FO was entirely replaced by LO. Similarly, the hyperlipidemic effect caused by dietary LO was also observed in black seabream (*Acanthopagrus schlegelii*) (Jin et al., 2017), and Manchurian trout (*Brachymystax lenok*) (Yu et al., 2019). Additionally, OA and LNA could increase serum lipid content, while n-3 LC-PUFA could reduce serum lipid content (Vegusdal et al., 2005; Hu et al., 2011; Shearer et al., 2012). In this study, an increase in the percentages of OA and LNA and a decrease in the percentage of n-3 LC-PUFA were found in diets with increasing LO levels. Therefore, the hyperlipemia of fish could be due to the imbalance of FAs in the 100% LO diet. However, different results were also observed that dietary LO decreased contents of serum TC and TG in yellow drum (Qin et al., 2020), hybrid sturgeon (*Acipenser baeri* Brandt♀ × *A. schrenckii* Brandt♂) (Liu et al., 2018), and largemouth bass (*Micropterus salmoides*) (Shi C. M. et al., 2019). Those different results might be related to the culture condition, feed ingredients or species differences in experiments. However, the specific reason remains to be further explored in the future. Since the liver is the central organ for lipid metabolism, liver damage is usually accompanied by increased ALT and AST activity in the serum (Giannini et al., 1999). When dietary FO was entirely replaced by LO, *L. crocea* had the higher activity of ALT in the serum than the control group. Similar results were found in turbot (Wang et al., 2016), and black seabream (Jin et al., 2017). In this study, the increased activity of ALT in the serum indicated that a high level of LO might cause some damage in the liver of fish.

Antioxidant capacity was also an important term in evaluating the physiological state of fish. CAT was a type II detoxifying enzyme that played a vital role in scavenging toxic intermediates (Samanta et al., 2014), while MDA was commonly used as an index to evaluate lipid peroxidation, which could indirectly reflect the degree of cell impairment (Mendes et al., 2009). The current study reported that *L. crocea* fed the diet with 100% LO had lower CAT activity and a higher concentration of MDA than the control group. Similar effects were found in Japanese seabass (*Lateolabrax japonicus*) (Tan et al., 2017) and hybrid grouper (*Epinephelus fuscoguttatus* ♀ × *E. lanceolatus* ♂) (An et al., 2020). ALA has been confirmed to increase mitochondrial DNA methylation in the liver, which can induce oxidative stress in fish (Liao et al., 2015). Meanwhile, EPA and DHA have been confirmed to relieve oxidative stress-related mitochondrial dysfunction and can improve activities of type II detoxifying enzymes (Oppedisano et al., 2020). According to these studies, the decrease of antioxidant capacity in *L. crocea* could be due to the increased content of ALA and the decreased contents of EPA and DHA in the 100% LO diet.

In this study, the total substitution of dietary FO with LO increased the hepatic lipid content of *L. crocea*. Similarly, dietary LO increased the abnormal lipid deposition in rainbow trout (Turchini and Francis, 2009), Japanese seabass (Xu et al., 2015), tilapia (Li et al., 2016), and yellow drum (Qin et al., 2020). The increase of abnormal lipid deposition was probably due to the increased percentage of ALA in the 100% LO diet, which could alter lipid metabolism. Previous studies have shown that FATP1 and CD36 are vital FA transport proteins in the liver (Stahl, 2004; Musso et al., 2009). In this study, when dietary FO was entirely replaced by LO, *L. crocea* had higher transcriptional levels of *fatp1* and *cd36* and a higher expression of the CD36 protein than the control group. A previous study have showed that the uptake of PUFA was dependent on membrane proteins in Atlantic salmon hepatocytes, where the uptake process was on this ranking list: EPA > ALA = DHA > LNA > OA (Zhou et al., 2010). Although the percentage of n-3 LC-PUFA in the 100% LO diet was lower than that in the FO diet, the percentage of ALA in the 100% LO diet was much higher than the percentage of n-3 LC-PUFA in the FO diet. The increased transcriptional levels of *fatp1* and *cd36* and expression of the CD36 protein were probably due to the high amount of the ALA in the 100% LO diet. Furthermore, CPT-1 is considered a significant enzyme in the FA oxidation pathway (Mcgarry and Brown, 2010). In this study, when dietary FO was entirely replaced by LO, fish had lower *cpt-1* mRNA expression than the control group. Moreover, dietary perilla oil (rich in ALA) was reported to increase the methylation of mitochondrial DNA in *L. crocea* which could cause the depression in FA oxidation (Liao et al., 2015). The decrease of *cpt-1* mRNA expression could be due to the excessive ALA in the 100% LO diet that might increase the mitochondrial DNA methylation. Therefore, the increase of lipid deposition in *L. crocea* fed the diet with 100% LO could be attributed to the up-regulation in FA uptake and the down-regulation in FA oxidation.

As abnormal lipid deposition was associated with chronic inflammation in fish (Tan et al., 2017), we investigated whether the inflammation was related to the negative physiological status of *L. crocea*. When dietary FO was entirely replaced by LO, fish had higher transcriptional levels of pro-inflammatory genes (*il-6*, *il-1*β, and *tnf*α) but lower transcriptional levels of anti-inflammatory genes (*arg1 and tgf*β) than the control group. Similarly, total substitution dietary FO with LO induced inflammation in yellow drum (Qin et al., 2020), and gilthead seabream (Montero et al., 2010). The inflammation caused by a high level of dietary LO was most likely attributed to the imbalance of dietary FAs. Although the 100% LO diet contained enough ALA, *L. crocea* had a limited capacity to synthesize EPA and DHA (essential FAs for marine fish) from C18 PUFA (Tocher, 2010; Li et al., 2017). The limited capacity and low n3 LC-PUFA content in the 100% LO diet would result in an unsatisfied amount of essential FA for *L. crocea* to maintain positive inflammatory responses.

## Conclusion

In conclusion, no more than 66.7% of FO can be substituted with LO without significantly decreasing the growth performance of large yellow croaker. However, high levels of dietary LO caused growth reduction, abnormal lipid deposition, and inflammation in the liver of fish. In addition, the increase of hepatic lipid deposition caused by 100% LO was probably attributed to the increase of fatty acid uptake and the decrease of fatty acid oxidation in fish.

## Data Availability Statement

The original contributions presented in the study are included in the article/supplementary file, further inquiries can be directed to the corresponding author/s.

## Ethics Statement

The animal study was reviewed and approved by the Standard Operation Procedures (approval number: 20001001) of the Institutional Animal Care and Use Committee of the Ocean University of China.

## Author Contributions

KM, QA, and XL designed the research. XL, QC, and KC conducted the research. XL, QL, and JL analyzed the data. XL wrote the manuscript. XL, YZ, and AK provided language help. All authors reviewed and approved the final manuscript.

## Conflict of Interest

The authors declare that the research was conducted in the absence of any commercial or financial relationships that could be construed as a potential conflict of interest.

## References

[B1] Ai Q. H. Mai K. S. Tan B. P. Xu W. Duan Q. Y. Ma H. M. (2006). Replacement of fish meal by meat and bone meal in diets for large yellow croaker. *Pseudosciaena crocea*. *Aquaculture* 260 255–263. 10.1016/j.aquaculture.2006.06.043

[B2] An W. Dong X. Tan B. Wu M. Zhang S. Chi S. (2020). Effects of dietary vegetable oil on growth performance, digestive capacity, antioxidant capacity and expression of immune-related genes in the hybrid grouper (*Epinephelus fuscoguttatus* ♀× *E. lanceolatus* ♂). *Aquacult. Nutr.* 26 2086–2101. 10.1111/anu.13149

[B3] Association of Official Analytical Chemists (AOAC), (1995). *Official methods of analysis of AOAC international*, 16th Edn. Arlington, VA: Association of Official Analytical Chemists.

[B4] Berg D. J. Leach M. W. Kühn R. Rajewsky K. Müller W. Davidson N. J. (1995). Interleukin 10 but not interleukin 4 is a natural suppressant of cutaneous inflammatory responses. *J. Exp. Med.* 182 99–108. 10.1084/jem.182.1.99 7790826 PMC2192105

[B5] Chen S. Su Y. Hong W. (2018). Aquaculture of the large yellow croaker. *Aquacult. China* 2018 297–308. 10.1002/9781119120759.ch3_10

[B6] Cui K. Li Q. F. Xu D. Zhang J. Z. Gao S. N. Xu W. (2020). Establishment and characterization of two head kidney macrophage cell lines from large yellow croaker (*Larimichthys crocea*). *Dev. Comp. Immunol.* 102:103477. 10.1016/j.dci.2019.103477 31470020

[B7] Eberlé D. Hegarty B. Bossard P. Ferré P. Foufelle F. (2004). SREBP transcription factors: master regulators of lipid homeostasis. *Biochimie* 86 839–848. 10.1016/j.biochi.2004.09.018 15589694

[B8] Feng S. H. Cai Z. N. Zuo R. T. Mai K. S. Ai Q. H. (2017). Effects of dietary phospholipids on growth performance and expression of key genes involved in phosphatidylcholine metabolism in larval and juvenile large yellow croaker, *Larimichthys crocea*. *Aquaculture* 469 59–66. 10.1016/j.aquaculture.2016.12.002

[B9] Filiz K. Ahmet Necdet S. Ercüment A. Feyza, Ýçoðlu A. Abdullah T. (2017). Effect of dietary fish oil replacement with plant oils on growth performance and gene expression in juvenile rainbow trout (*Oncorhynchus mykiss*). *Ann. Anim. Sci.* 17 1135–1153. 10.1515/aoas-2017-0010

[B10] Folch J. Lees M. Stanley H. (1957). A simple method for the isolation and purification of total lipides from animal tissues. *J. Biol. Chem.* 226 497–509. 13428781

[B11] Giannini E. Botta F. Fasoli A. Ceppa P. Risso D. Lantieri P. B. (1999). Progressive liver functional impairment is associated with an increase in AST/ALT ratio. *Dig. Dis. Sci.* 44 1249–1253. 10.1023/A:1026609231094 10389705

[B12] He Y. Chi S. Tan B. Dong X. Yang Q. Liu H. (2019). dl-Methionine supplementation in a low-fishmeal diet affects the TOR/S6K pathway by stimulating ASCT2 amino acid transporter and insulin-like growth factor-I in the dorsal muscle of juvenile cobia (*Rachycentron canadum*). *Brit. J. Nutr.* 122 734–744. 10.1017/S0007114519001648 32124713

[B13] Hu Y. Tan B. P. Mai K. S. Ai Q. H. Zhang L. Zheng S. (2011). Effects of dietary menhaden oil, soybean oil and soybean lecithin oil at different ratios on growth, body composition and blood chemistry of juvenile *Litopenaeus vannamei*. *Aquacult. Int.* 19 459–473. 10.1007/s10499-010-9361-4

[B14] Huang W. Yao C. Liu Y. Xu N. Yin Z. Xu W. (2020). Dietary allicin improved the survival and growth of large yellow croaker (*Larimichthys crocea*) larvae via promoting intestinal development, alleviating inflammation and enhancing appetite. *Front. Physiol.* 11:587674. 10.3389/fphys.2020.587674 33162901 PMC7583326

[B15] Hussain M. M. Rava P. Walsh M. Rana M. Iqbal J. (2012). Multiple functions of microsomal triglyceride transfer protein. *Nutr. Metab.* 9:14. 10.1186/1743-7075-9-14 22353470 PMC3337244

[B16] Jin M. Yuan Y. Lu Y. Ma H. Sun P. Li Y. (2017). Regulation of growth, tissue fatty acid composition, biochemical parameters and lipid related genes expression by different dietary lipid sources in juvenile black seabream, *Acanthopagrus schlegelii*. *Aquaculture* 479 25–37. 10.1016/j.aquaculture.2017.05.017

[B17] Kühn R. Löhler J. Rennick D. Rajewsky K. Müller W. (1993). Interleukin-10-deficient mice develop chronic enterocolitis. *Cell* 75 263–274. 10.1016/0092-8674(93)80068-P 8402911

[B18] Li F. J. Lin X. Lin S. M. Chen W. Y. Guan Y. (2016). Effects of dietary fish oil substitution with linseed oil on growth, muscle fatty acid and metabolism of tilapia (*Oreochromis niloticus*). *Aquacult. Nutr.* 22 499–508. 10.1111/anu.12270

[B19] Li J. M. Li L. Y. Qin X. Degrace P. Demizieux L. Limbu S. M. (2018). Inhibited carnitine synthesis causes systemic alteration of nutrient metabolism in zebrafish. *Front. Physiol.* 9:509. 10.3389/fphys.2018.00509 29867554 PMC5954090

[B20] Li S. L. Li Z. Q. Zhang J. C. Sang C. Y. Chen N. S. (2019). The impacts of dietary carbohydrate levels on growth performance, feed utilization, glycogen accumulation and hepatic glucose metabolism in hybrid grouper (*Epinephelus fuscoguttatus* ♀× *E. lanceolatus* ♂). *Aquaculture* 512:734351. 10.1016/j.aquaculture.2019.734351

[B21] Li S. L. Monroig Ó Wang T. J. Yuan Y. H. Carlos Navarro J. Hontoria F. (2017). Functional characterization and differential nutritional regulation of putative Elovl5 and Elovl4 elongases in large yellow croaker (*Larimichthys crocea*). *Sci. Rep.* 7:2303. 10.1038/s41598-017-02646-8 28536436 PMC5442133

[B22] Li X. S. Ji R. L. Cui K. Chen Q. Chen Q. C. Fang W. (2019). High percentage of dietary palm oil suppressed growth and antioxidant capacity and induced the inflammation by activation of TLR-NF-κB signaling pathway in large yellow croaker (*Larimichthys crocea*). *Fish Shellf. Immun.* 87 600–608. 10.1016/j.fsi.2019.01.055 30738147

[B23] Li Y. N. Pang Y. N. Xiang X. J. Du J. L. Mai K. S. Ai Q. H. (2019). Molecular cloning, characterization, and nutritional regulation of Elovl6 in large yellow croaker (*Larimichthys crocea*). *Int. J. Mol. Sci.* 20:20071801. 10.3390/ijms20071801 30979053 PMC6480403

[B24] Liao K. Yan J. Mai K. S. Ai Q. H. (2015). Dietary olive and perilla oils affect liver mitochondrial DNA methylation in large yellow croakers. *J. Nutr.* 145 2479–2485. 10.3945/jn.115.216481 26400965

[B25] Liu C. Wang J. Ma Z. Li T. Xing W. Jiang N. (2018). Effects of totally replacing dietary fish oil by linseed oil or soybean oil on juvenile hybrid sturgeon, *Acipenser baeri* Brandt♀×*A. schrenckii* Brandt♂. *Aquacult. Nutr.* 24 184–194. 10.1111/anu.12546

[B26] Livak K. J. Schmittgen T. D. (2001). Analysis of relative gene expression data using real-time quantitative PCR and the 2(-Delta Delta C(T)) Method. *Methods* 25 402–408. 10.1006/meth.2001 11846609

[B27] Mai K. S. Wan J. Ai Q. H. Xu W. Liufu Z. G. Zhang L. (2006). Dietary methionine requirement of large yellow croaker, *Pseudosciaena crocea* R. *Aquaculture* 253 564–572. 10.1016/j.aquaculture.2005.08.010

[B28] Mcgarry J. D. Brown N. F. (2010). The mitochondrial carnitine palmitoyltransferase system. From concept to molecular analysis. *FEBS J.* 244 1–14. 10.1111/j.1432-1033.1997.00001.x 9063439

[B29] Mendes R. Cardoso C. Pestana C. (2009). Measurement of malondialdehyde in fish: A comparison study between HPLC methods and the traditional spectrophotometric test. *Food Chem.* 112 1038–1045. 10.1016/j.foodchem.2008.06.052

[B30] Menendez J. A. Lupu R. (2007). Fatty acid synthase and the lipogenic phenotype in cancer pathogenesis. *Nat. Rev. Cancer* 7 763–777. 10.1038/nrc2222 17882277

[B31] Menoyo D. Izquierdo M. S. Robaina L. Ginés R. Lopez-Bote C. J. Bautista J. M. (2004). Adaptation of lipid metabolism, tissue composition and flesh quality in gilthead sea bream (*Sparus aurata*) to the replacement of dietary fish oil by linseed and soyabean oils. *Brit. J. Nutr.* 92 41–52. 10.1079/BJN20041165 15230986

[B32] Menoyo D. Lopez-Bote C. J. Diez A. Obach A. Bautista J. M. (2007). Impact of n-3 fatty acid chain length and n-3/n-6 ratio in Atlantic salmon (*Salmo salar*) diets. *Aquaculture* 267 248–259. 10.1016/j.aquaculture.2007.02.031

[B33] Metcalfe L. D. Schmitz A. A. Pelka J. R. (1966). Rapid preparation of fatty acid esters from lipids for gas chromatographic analysis. *Anal. Chem.* 38 514–515. 10.1021/ac60235a044

[B34] Montero D. Grasso V. Izquierdo M. S. Ganga R. Real F. Tort L. (2008). Total substitution of fish oil by vegetable oils in gilthead sea bream (*Sparus aurata*) diets: Effects on hepatic Mx expression and some immune parameters. *Fish Shellf. Immun.* 24 147–155. 10.1016/j.fsi.2007.08.002 18158252

[B35] Montero D. Mathlouthi F. Tort L. Afonso J. M. Torrecillas S. Fernández-Vaquero A. (2010). Replacement of dietary fish oil by vegetable oils affects humoral immunity and expression of pro-inflammatory cytokines genes in gilthead sea bream *Sparus aurata*. *Fish Shellf. Immun.* 29 1073–1081. 10.1016/j.fsi.2010.08.024 20817101

[B36] Mu H. Wei C. Q. Zhang Y. J. Zhou H. H. Pan Y. Chen J. (2020). Impacts of replacement of dietary fish oil by vegetable oils on growth performance, anti-oxidative capacity, and inflammatory response in large yellow croaker *Larimichthys crocea*. *Fish Physiol. Biochem.* 46 231–245. 10.1007/s10695-019-00712-8 31734894

[B37] Musso G. Gambino R. Cassader M. (2009). Recent insights into hepatic lipid metabolism in non-alcoholic fatty liver disease (NAFLD). *Prog. Lipid. Res.* 48 1–26. 10.1016/j.plipres.2008.08.001 18824034

[B38] Nayak M. Saha A. Pradhan A. Samanta M. Giri S. S. (2017). Dietary fish oil replacement by linseed oil: Effect on growth, nutrient utilization, tissue fatty acid composition and desaturase gene expression in silver barb (*Puntius gonionotus*) fingerlings. *Comp. Biochem. Phys. B* 205 1–12. 10.1016/j.cbpb.2016.11.009 27913275

[B39] Olofsson S. O. BorÈN J. (2005). Apolipoprotein B: a clinically important apolipoprotein which assembles atherogenic lipoproteins and promotes the development of atherosclerosis. *J. Intern. Med.* 258 395–410. 10.1111/j.1365-2796.2005.01556.x 16238675

[B40] Oppedisano F. Macrì R. Gliozzi M. Musolino V. Mollace V. (2020). The anti-inflammatory and antioxidant properties of n-3 PUFAs: their role in cardiovascular protection. *Biomedicines* 8 1–18. 10.3390/biomedicines8090306 32854210 PMC7554783

[B41] Peng X. Li F. Lin S. Chen Y. (2016). Effects of total replacement of fish oil on growth performance, lipid metabolism and antioxidant capacity in tilapia (*Oreochromis niloticus*). *Aquacult. Int.* 24 145–156. 10.1007/s10499-015-9914-7

[B42] Pohlenz C. Gatlin D. M. (2014). Interrelationships between fish nutrition and health. *Aquaculture* 431 111–117. 10.1016/j.aquaculture.2014.02.008

[B43] Qin G. Xu D. Lou B. Chen R. Wang L. Tan P. (2020). iTRAQ-based quantitative phosphoproteomics provides insights into the metabolic and physiological responses of a carnivorous marine fish (*Nibea albiflora*) fed a linseed oil-rich diet. *J. Proteomics* 228:103917. 10.1016/j.jprot.2020.103917 32738521

[B44] Ruyter B. Andersen Ø Dehli A. Östlund Farrants K. Gjøen T. Thomassen M. S. (1997). Peroxisome proliferator activated receptors in Atlantic salmon (*Salmo salar*): effects on PPAR transcription and acyl-CoA oxidase activity in hepatocytes by peroxisome proliferators and fatty acids. BBA-Lipids. *Lipid. Metabol.* 1348 331–338. 10.1016/S0005-2760(97)00080-5 9366249

[B45] Samanta P. Pal S. Mukherjee A. K. Ghosh A. R. (2014). Biochemical effects of glyphosate based herbicide, Excel Mera 71 on enzyme activities of acetylcholinesterase (AChE), lipid peroxidation (LPO), catalase (CAT), glutathione-S-transferase (GST) and protein content on teleostean fishes. *Ecotox. Environ. Safe* 107 120–125. 10.1016/j.ecoenv.2014.05.025 24927388

[B46] Shearer G. C. Savinova O. V. Harris W. S. (2012). Fish oil-How does it reduce plasma triglycerides? *BBA Mol. Cell Bio. L* 1821 843–851. 10.1016/j.bbalip.2011.10.011 22041134 PMC3563284

[B47] Shi C. M. Zhao H. Zhai X. L. Chen Y. J. Lin S. M. (2019). Linseed oil can decrease liver fat deposition and improve antioxidant ability of juvenile largemouth bass, Micropterus salmoides. *Fish Physiol. Biochem.* 45 1513–1521. 10.1007/s10695-019-00636-3 30945042

[B48] Shi Y. Zhong L. Ma X. Liu Y. Tang T. Hu Y. (2019). Effect of replacing fishmeal with stickwater hydrolysate on the growth, serum biochemical indexes, immune indexes, intestinal histology and microbiota of rice field eel (*monopterus albus*). *Aquacult. Rep.* 15:100223. 10.1016/j.aqrep.2019.100223

[B49] Stahl A. (2004). A current review of fatty acid transport proteins (SLC27). *Pflügers Archiv.* 447 722–727. 10.1007/s00424-003-1106-z 12856180

[B50] Tan P. Dong X. J. Xu H. L. Mai K. S. Ai Q. H. (2017). Dietary vegetable oil suppressed non-specific immunity and liver antioxidant capacity but induced inflammatory response in Japanese sea bass (*Lateolabrax japonicus*). *Fish Shellf. Immun.* 63 139–146. 10.1016/j.fsi.2017.02.006 28189766

[B51] Tan X. Y. Luo Z. Xie P. Liu X. J. (2009). Effect of dietary linolenic acid/linoleic acid ratio on growth performance, hepatic fatty acid profiles and intermediary metabolism of juvenile yellow catfish *Pelteobagrus fulvidraco*. *Aquaculture* 296 96–101. 10.1016/j.aquaculture.2009.08.001

[B52] Tocher D. R. (2010). Fatty acid requirements in ontogeny of marine and freshwater fish. *Aquac. Res.* 41 717–732. 10.1111/j.1365-2109.2008.02150.x

[B53] Turchini G. M. Francis D. S. (2009). Fatty acid metabolism (desaturation, elongation and β-oxidation) in rainbow trout fed fish oil- or linseed oil-based diets. *Brit. J. Nutr.* 102 69–81. 10.1017/S0007114508137874 19123959

[B54] Turchini G. M. Francis D. S. Senadheera S. P. S. D. Thanuthong T. De Silva S. S. (2011). Fish oil replacement with different vegetable oils in Murray cod: Evidence of an “omega-3 sparing effect” by other dietary fatty acids. *Aquaculture* 315 250–259. 10.1016/j.aquaculture.2011.02.016

[B55] Turchini G. M. Torstensen B. E. Ng W. K. (2009). Fish oil replacement in finfish nutrition. *Rev. Aquacult.* 1 10–57. 10.1111/j.1753-5131.2008.01001.x

[B56] Uhlen M. Bandrowski A. Carr S. Edwards A. Ellenberg J. Lundberg E. (2016). A proposal for validation of antibodies. *Nat. Methods* 13 823–827. 10.1038/nmeth.3995 27595404 PMC10335836

[B57] Vegusdal A. Gjøen T. Berge R. K. Thomassen M. S. Ruyter B. (2005). Effect of 18:1n-9, 20:5n-3, and 22:6n-3 on lipid accumulation and secretion by atlantic salmon hepatocytes. *Lipids* 40 477–486. 10.1007/s11745-005-1407-z 16094857

[B58] Wabike E. E. Wu X. Zhu W. Lou B. Chen R. Xu D. (2020). Partial replacement of fish oil with terrestrial lipid blend and effects on growth performance, body composition, immune parameter and growth-related genes in yellow drum (*Nibea albiflora*). *Aquacult. Nutr.* 26 954–963. 10.1111/anu.13053

[B59] Wang Q. C. He G. Mai K. S. (2016). Modulation of lipid metabolism, immune parameters, and hepatic transferrin expression in juvenile turbot (*Scophthalmus maximus* L.) by increasing dietary linseed oil levels. *Aquaculture* 464 489–496. 10.1016/j.aquaculture.2016.07.030

[B60] Wu F. C. Chen H. Y. (2012). Effects of dietary linolenic acid to linoleic acid ratio on growth, tissue fatty acid profile and immune response of the juvenile grouper *Epinephelus malabaricus*. *Aquaculture* 32 111–117. 10.1016/j.aquaculture.2011.10.030

[B61] Xiao S. Han Z. Wang P. Han F. Liu Y. Li J. (2015). Functional marker detection and analysis on a comprehensive transcriptome of large yellow croaker by next generation sequencing. *PLoS One* 10:e0124432. 10.1371/journal.pone.0124432 25909910 PMC4409302

[B62] Xu D. He G. Mai K. Zhou H. Xu W. Song F. (2016). Postprandial nutrient-sensing and metabolic responses after partial dietary fishmeal replacement by soyabean meal in turbot (*Scophthalmus maximus* L.). *Brit. J. Nutr.* 115 379–388. 10.1017/S0007114515004535 26586314

[B63] Xu H. G. Zhang Y. J. Wang J. Zuo R. T. Mai K. S. Ai Q. H. (2015). Replacement of fish oil with linseed oil or soybean oil in feeds for Japanese Seabass, *Lateolabrax japonicus*: Effects on growth performance, immune response, and tissue fatty acid composition. *J. World Aquacult. Soc.* 46 349–362. 10.1111/jwas.12205

[B64] Yan J. Liao K. Wang T. J. Mai K. S. Xu W. Ai Q. H. (2015). Dietary lipid levels influence lipid deposition in the liver of large yellow croaker (*Larimichthys crocea*) by regulating lipoprotein receptors, fatty acid uptake and triacylglycerol synthesis and catabolism at the transcriptional level. *PLoS One* 10:e0129937. 10.1371/journal.pone.0129937 26114429 PMC4482732

[B65] Yu J. Li S. Niu H. Chang J. Hu Z. Han Y. (2019). Influence of dietary linseed oil as substitution of fish oil on whole fish fatty acid composition, lipid metabolism and oxidative status of juvenile Manchurian trout. *Sci. Rep.* 9 1–10. 10.1038/s41598-019-50243-8 31554849 PMC6761147

[B66] Zeng Y. Y. Jiang W. D. Liu Y. Wu P. Zhao J. Jiang J. (2016). Optimal dietary alpha-linolenic acid/linoleic acid ratio improved digestive and absorptive capacities and target of rapamycin gene expression of juvenile grass carp (*Ctenopharyngodon idellus*). *Aquacult. Nutr.* 22 1251–1266. 10.1111/anu.12337

[B67] Zheng J. L. Zeng L. Shen B. Xu M. Y. Zhu A. Y. Wu C. W. (2016). Antioxidant defenses at transcriptional and enzymatic levels and gene expression of Nrf2-Keap1 signaling molecules in response to acute zinc exposure in the spleen of the large yellow croaker *Pseudosciaena crocea*. *Fish Shellf. Immun.* 52 1–8. 10.1016/j.fsi.2016.02.031 26940795

[B68] Zhou J. Stubhaug I. Torstensen B. E. (2010). Trans-Membrane Uptake and Intracellular Metabolism of Fatty Acids in Atlantic Salmon (*Salmo salar* L.) Hepatocytes. *Lipids* 45 301–311. 10.1007/s11745-010-3396-1 20186497

[B69] Zou J. Secombes C. J. (2016). The function of fish cytokines. *Biology* 5 1–35. 10.3390/biology5020023 27231948 PMC4929537

[B70] Zuo R. T. Ai Q. H. Mai K. S. Xu W. Wang J. Xu H. G. (2012). Effects of dietary n-3 highly unsaturated fatty acids on growth, nonspecific immunity, expression of some immune related genes and disease resistance of large yellow croaker (*Larmichthys crocea*) following natural infestation of parasites (*Cryptocaryon irritans*). *Fish Shellf. Immun.* 32 249–258. 10.1016/j.fsi.2011.11.005 22126857

